# Relationship between self‐esteem and perceived social support in burn patients in Sina Hospital of Tabriz

**DOI:** 10.1002/nop2.734

**Published:** 2020-12-09

**Authors:** Azam Gorbani, Monireh Rezaiee Moradali, Reza Shabanloei

**Affiliations:** ^1^ Department of Nursing and Midwifery, Urmia Branch Islamic Azad University Urmia Iran; ^2^ Department of Medical Surgical Nursing Faculty of Nursing and Midwifery Sina Hospital Tabriz University of Medical Sciences Tabriz Iran

**Keywords:** burns, nurses, nursing, self‐esteem, social support

## Abstract

**Aim:**

Visible scars and damage to self‐esteem make it difficult to endure difficult conditions and have some detrimental psychological and physical consequences for patients with burns. Social support plays an important role in enhancing the mental image and self‐esteem of burn patients in the rehabilitation stages of burn patients. The aim of this study was to determine the relationship between perceived social support and self‐esteem in burn patients referred to Sina Hospital in Tabriz in 2019–2018.

**Design:**

Descriptive correlational study.

**Methods:**

In this descriptive correlational study, 120 patients with different degrees of burn were selected using the convenience sampling method. After obtaining validity and reliability, the data were collected using demographic questionnaires, Rosenberg self‐esteem and Zimet Multi‐dimensional Perceived Social Support. Then, they were analysed using descriptive and analytical tests and SPSS software.

**Results:**

There was a positive and significant correlation between the main variables of the research (*p* < .001, *r* = 0.288). Duration of hospitalization, percentage of burns and education were effective factors in self‐esteem (*p* < .005). Based on the findings, more support for burn patients will increase patients' self‐esteem and greater adaptation, as well as a better return to their lives in the rehabilitation phase.

## 1 INTRODUCTION

Burn injuries have been defined as damages caused by the application of heat, chemicals and electrical current, or radiation to the outer or inner surface of the body, causing tissue depletion (Ganesamoni et al., 2010). Burns are one of the most devastating injuries. In terms of prevalence after traffic accidents, crashes and interpersonal violence, they are the fourth type of injuries (Zakeri et al., 2018).

Burns on the head, neck and face can be seen as scars. Scars and contractures are the most common complications after treatment for burn injuries. Scars in visible areas are associated with social anxiety, isolation and poor social quality (Hoogewerf et al., 2014) and can result in permanent mental and psychological changes (Ilechukwu, 2002).

Annually, in Iran, 150,000 burns occurred each year, with an annual death of 3,000 individuals (Dehghani et al., 2014; Vaghardoost et al., 2016). With increasing survival rate among burn patients, it is important to manage the psychological needs of burn survivors to achieve the quality of life and performance close to the previous burn level and successful survivors' integration into society with a healthy mind and body (Falder et al., 2009).

Significant burn scars may lead to social exclusion by affecting individual behaviours (Pope et al., 2007). Understanding social support can prevent undesirable physiological complications and increase the level of self‐care and have a positive effect on the physical, mental, psychological and spiritual condition of the person and ultimately lead to improved performance (Farahani et al., 2017).

Survivors of burns have reported greater dissatisfaction with their appearance and lower mood and lower quality of life (Pope et al., 2007). Unpleasant physical appearance can have a negative effect on a person's self‐esteem (Fiske, 2010). Burn injuries are a distressing experience that can lead to disability and a pessimistic impact on different aspects of life (Ciofi‐Silva et al., 2010). One of the factors that plays an important role in mental health is having self‐esteem. Decreased self‐esteem can be detrimental to people's health (von Soest et al., 2017).

Rosenberg defines self‐esteem as: a sense of self‐sufficiency to face the fundamental challenges of life and to be worthy of happiness. Self‐esteem stems from the difference between the self‐perceived and the ideal self. The greater the difference between the two, the lower the self‐esteem (Mruk, 2006).

Undoubtedly, one of the important reasons for researchers' attention to the concept of self‐esteem is its potential impact on health. It has been proven that self‐esteem damage makes it impossible to endure the difficult conditions that people inevitably face in daily life and has detrimental psychological and physical consequences for them (Ferreira et al., 2017; Zuckerman, 1989).

In the study of Aklechi et al found that low self‐esteem led to behaviours such as decreased performance, inadequacy, loneliness and self‐destructive behaviours (Aklechi, 2012).On the other hand, many social and cultural criteria influence the evolution of the mental image such as the way we deal with community, family and friends (Sideli et al., 2010).

Psychological effects of burn outcomes are debilitating and require constant rehabilitation of the mental image in the course of the disease. More than 40% of burn patients suffer from mental disorders in the first 2 years after burn and people with less social support feel more stress and pain (Sideli et al., 2010).

After the first years of rehabilitation of burn survivors, social support is directly related to satisfaction with appearance, mental image and self‐esteem (Ajoudani et al., 2018). Social support such as having an effective network of friends and family can be effective in one's health care (Farahani et al., 2017).

Since low self‐esteem in patients with burn injuries has a negative effect on interpersonal relationships and patients' thinking, feelings and performance, it is important to evaluate it in these patients (Sharifi Neyestanak et al., 2012).

Although much research has been done on social support and self‐esteem; however, after reviewing the literature of electronic resources in our country, it was found that regarding social support and self‐esteem in patients with burns, little research has been done. Therefore, according to the researcher's experiences, it seems that burn patients have less social support and this issue can reduce the self‐esteem of burn patients. Accordingly, this study aimed to investigate the relationship between self‐esteem and social support in patients with burns.

## 2 MATERIALS AND METHODS

### 2.1 Design of the study

This is a descriptive correlational research conducted during the six months from March 2018 to the end of August 2019.

### 2.2 Setting and participants

The study population consisted of burn patients admitted to burns wards and outpatients at Sina hospital in Tabriz. Study participants were selected from patients who came to the clinic to follow the treatment process of the consequences and complications of burns as well as patients who were hospitalized for heal burns or cosmetic surgery following burns. Inclusion criteria included burns in visible areas, reading literacy, visible scars, at least one month of burns and exclusion criteria were unwillingness to interview, patients with underlying mental problems such as self‐harm, incomplete questionnaire filling of 10%–15%. Patients with burns, due to the different nature of the injury and physical deformity, in addition to the physical and psychological dimension, also suffer from the social dimension. Therefore, in this study, we considered all patients with burns in visible areas as participants.

This study was conducted at Sina Tabriz hospital which is known as the Northwest Burns Referral Center of Iran. Sina hospital with 78 active beds for patients (the women's burn ward has 19 beds, the men's and paediatric burn ward each has 18 beds, reconstruction wards has 14 beds and burn intensive care unit (BICU) has 9 beds), clinic of burn and physiotherapy with admits approximately 1,200 burned patients annually. All burn rooms were designed as one bed and in accordance with international standards. The families speak Persian, Azerbaijani and Turkish.

After the pilot study on 40 patients and considering the correlation coefficient of 0.14 and 1.96 α and power of 0.8 after applying the following formula, the sample size required was at least 79 patients. For accuracy, given that sampling was carried out in several stages, one and a half times the sample size of 120 was considered for the study. These 120 participants were selected by the convenience sampling method among the patients referred to the clinic and hospitalized patients based on inclusion and exclusion criteria:

The correlation coefficient of confidence → $\omega =\frac{1}{2}Ln\frac{1+r}{1‐r}$

$$n=\frac{{\left(Z_{1‐\frac{\alpha }{2}}+Z_{1‐\beta }\right)}^{2}}{{\left(\omega \right)}^{2}}+3.$$

### 2.3 Description of instrumentation

To collect information, a three‐part questionnaire was used that including: Demographic data, Persian version of Zimet social support questionnaire and Rosenberg self‐esteem questionnaire.

Personal information form including age, gender, marital status, degree of burn (as determined by the physician and recorded in the patient's baseline assessment sheet), burn percentage, education level, location of burn, cause of burn, language, religion, length of hospitalization, economic status, job and time of burn.

The Rosenberg questionnaire was used to assess the self‐esteem of burn patients in this study. The Rosenberg Self‐Esteem Questionnaire (RSES) developed it in 1965. The questionnaire consists of ten items that measure a person's positive and negative feelings about himself or herself. In Iran, the second form of the questionnaire has been prepared and scored as *I agree and I disagree* (Greenberger et al., 2003; Huis et al., 2011). Scoring was such that the answer *I agree* to each of the 1–5 expressions would receive + 1, the answer *I disagree* to each of the 1–5 expressions would receive −1. The answer *I agree* to each of the 6–10 expressions would receive −1, the answer *I disagree* to each of the 6–10 expressions would receive +1. A score above zero indicates high self‐esteem, a score below zero indicates low self‐esteem and a score of −10 indicates very low self‐esteem.

The Multi‐dimensional Perceived Social Support Scale (MSPSS) was used to assess the Social Support of burn patients in this study. The MSPSS developed by Zimet et al. (1988) measured three sources of family, friends and other important individuals. There were 12 questions in this questionnaire. Questions 3, 4, 8, 11 measured family resource, the questions 6, 7, 9, 12 measured friends’ resource and the questions 1, 2, 5, 10 measured others support source. There were 5 responses for each expression, including I strongly disagree (1), I disagree (2), I have no opinion (3), I agree (4) and I strongly agree (5). Scores range is from 12–60; scores 12–20 show the perceived low level of social support. The scores 20–40 show the perceived moderate level of social support. A score above 40 means the perceived high level of social support (Behzadfar et al., 2018).

Face and content validity were used to determine the validity of the questionnaire. This tool was provided to 6 faculty members of Urmia Azad Faculty of Nursing & Midwifery and 4 experienced nurses caring for burns. In this research, we calculated Cronbach's alpha coefficient for reliability using 40 questionnaires. Cronbach's alpha for self‐esteem questionnaire was (0.84) and for Zimet questionnaires was 0.75.

### 2.4 Data collection and analysis

After obtaining the necessary authorization and approval from the Ethical Committee of the Faculty of Nursing and Midwifery of Urmia Azad University and Sina Hospital in Tabriz, the researcher explained the goals to the department head and head nurse. Before completing the questionnaire by the participants, their informed consent was obtained. Assured that the information was confidential, and the patient was voluntary, patients with inclusion criteria completed the questionnaire. Descriptive statistics, standard deviation, mean and Pearson correlation coefficients were used for data analysis for comparing the variables through the software SPSS 24. The significance level was considered to be *p* > .005. Also, the normality of the quantitative variables used in the analysis was investigated by the descriptive indices through the K‐S test.

## 3 RESULTS

### 3.1 Demographic data

The mean age of hospitalization was 35.4%, of which 60.8% were female and 39.2% were male. 69.2% of people had burned in some areas. 46.7% of patients had burned in open areas and 53.3% of patients had burned in closed areas. In this study, the highest rate of fire burns was 64.2% and the number of housekeepers with 23.5% was the highest. In terms of economic status, 43.2% were at an average level. The mean burn rate was 35.8% and the mean hospital stay was 24.48 (Table 1). Other variables are listed in Table 1.

**TABLE 1:** Mean and standard deviation of age, length of hospitalization and percentage of burns

Variable	*N*	%
Gender		
Female	73	60.8
Male	47	39.2
Education		
Elementary/Middle School	50	41.7
Diploma	46	38.3
Bachelor	17	14.2
Academic	7	5.8
Marital status		
Married	75	62.5
Single	31	25.8
Divorced	7	5.8
Widow	7	5.8
Religion		
Shia Islam	100	83.3
Sunni Islam	19	15.8
Other	1	0.8
Employment Status		
Housewife	39	23.5
Unemployed	10	8.3
Worker	15	12
Employee	24	20
Free job	28	23.3
Student	4	3.3
The ratio of income to spend		
Negative	38	31.7
Equals	43	35.8
Positive	39	32.5
Burn site		
Face	1	0.8
Upper limb	1	0.8
Lower limbs	4	3.3
Face and upper limb	14	11.7
Face and lower limb	1	0.8
Face and trunk	3	2.5
Upper and lower limb	9	7.5
Upper and trunk	4	3.3
More than three areas	83	69.2
Covering the burn area		
Covered	64	53.3
Open	56	46.7
Cause of burn		
Scald	18	15
Fire	83	68.4
Electric burn	6	5
Chemical	14	11.7

	*N*	Mean	*SD*
Percentage of burns	120	35.84	12.16
Duration of hospitalization	120	24.48	13.28
Age	120	35.4	12.38

Abbreviations: %, Percentage; *N*, Frequency; *SD*, Standard Deviation.

### 3.2 Perceived social support and self‐esteem data

The mean score of family social support was 21.81 with a standard deviation of 5.55 and a mean score of self‐esteem was 2.70 with a standard deviation of 4.95. There was a positive and significant correlation between the two main variables of the study (*r* = 0.25, *p* < .006) such that increased family social support increases self‐esteem.

There was also a positive and significant relationship between the mean score of self‐esteem and the mean score of friends' support, and the mean score of other people's support (Table 2). There was a significant positive correlation between the mean score of total self‐esteem and a mean score of total social support (*r* = 0.288, *p* < .001), so that increasing social support increased self‐esteem (Table 2). Also, there was no significant difference in comparing perceived social support score and self‐esteem between men and women (Table 3).

**TABLE 2:** Relationship between mean total self‐esteem score and mean total social support score

Variable	Mean	*SD*	Statistical test
Total social support	52/59	38/14	*p* = 0/001
Total self‐esteem	70/2	95/0	*r* = 0/288
Family support	81/21	55/5	*p *= /006
Self‐esteem	70/2	95/4	*r* = 0/25
Supporting others	20/39	5/50	*p* = 0/001
Self‐esteem	2/70	4/94	*r* = 0/289
Friends support	32/17	96/5	*p* = 0/033
Self‐esteem	69/2	95/4	*r* = 0/195

**TABLE 3:** Comparison of mean scores of self‐esteem and social support in two groups of men and women

Variable	Mean	*SD*	Statistical test
Self‐esteem			
Men	1.21	0.60	*t* = −1.38
Women	1.06	0.53	*p* = 0/064
Social support			
Men	58.70	15.95	*t* = −0.809
Women	60.86	15.08	*p* = 0/617

## 4 DISCUSSION

The overall purpose of this research was to investigate the relationship between perceived social support and self‐esteem in burns. The results of this research showed that two variables of perceived social support and self‐esteem have a positive and significant correlation. People with high social support had high self‐esteem. This study was in line with the results of the study of Budd on the correlation between social support and self‐esteem (Budd et al., 2009; Fujiwara et al., 2019).

In fact, people with burns should have greater social support from family, friends and others, so the role of social support in coping with burn complications and coping with the resulted stresses, especially low self‐esteem, seems to be greater. As reported in the results of the study done by Robab et al., entitled “Assessment of self‐esteem and depression in burn‐affected women,” the self‐esteem was related to family support and type of burn and there was a significant difference between family support and burn area (Rubab & Kalsoom, 2018).

Studies have shown that young burn patients who have good mental status and supportive social and economic, social and family support networks before burn appear to perform well after burn (Pope et al., 2007). Those who have higher social support are more satisfied with their mental image (Niroumand‐Zandi, et al., 2016). Ajoudani et al. (2018) have provided insight into the relation of social participation and social support with mental image among burn survivors in the early stages of rehabilitation.

Concerning the other aims of the research, the results showed that there was no significant difference between the two groups of men and women in terms of self‐esteem and social support; whereas in the study of Zahid et al. (2017) this is not the case. Social support, in this study, has been reported to be an important factor in improving patients' self‐esteem, which is consistent with the present study. Consistent with these findings, research has shown that low self‐esteem leads to feelings of failure, dissatisfaction with one's role, low interpersonal skills and poor social interactions (De Moor et al., 2019).

Explaining this relationship, it can be said that high self‐esteem increases personal abilities and self‐sufficiency and leads to life satisfaction. In Iranian culture, due to the emphasis on collectivist culture, the amount and quality of self‐esteem are highly dependent on relationships with others (Duprey et al., 2019). In Iranian culture, self‐esteem seems to be closely related to perceived social support, whether by family or friends or other people, which requires further research (Abu‐Kaf et al., 2019; Eisenbarth, 2019; Karatekin & Ahluwalia, 2020).

In the study conducted by Massoud et al., there was a significant relationship between men and married people with PTSD psychologically after burn (Masood et al., 2018). It seems that because of the culture and religion in Iran and because of the emotional attachment, men and women are appropriately socially well supported. Waqas et al. (2016) revealed the results of an inadequate pattern of social support in a group of burn patients in Pakistan. They stated that addressing these inequalities in support provision should be prioritized as part of the burn care programme.

The results of a study carried out by Mir Salaheddin et al indicated that public health and social support were related to self‐immolation. Therefore, linking individuals with society and groups and providing social and emotional support reduce suicide rates (Enayati et al., 2006).

A study done by Niroumand‐Zandi et al. (2016) points to a low mental image following high burn percentages.

Also, the percentage of burns with the duration of burns, especially facial burns, is significantly associated with social and psychological domains and quality of life, as a study has shown a decrease in social connection in these people (Martin et al., 2017) .In our study, on the percentage of burn, people with high percentages had low self‐esteem. There was a significant difference between burn percentage and self‐esteem, while there was no significant difference between it and social support. This difference may be due to physical changes and numerous mental disorders, including changes in the mental image.

Low self‐esteem found in burned patients is not out of the expectation; because studies have shown that the burn scars can have a negative effect on the patient's self‐esteem with its effect on his/her appearance (von Soest et al., 2017). Jane et al. refer to the severity of burn as a cause of low self‐esteem, which is consistent with the present study (Jain et al., 2017).

### 4.1 Study limitations

This study, like other studies, had limitations. These limitations included the following:

Using the convenience sampling method in this study, all burn patients do not have an equal chance to participate in this study. The mental state of the participants while filling out the questionnaire was influential on how they responded. To prevent confounding factors, the researcher collected data in different shifts and during the period when patients were mentally, psychologically and physically in favourable conditions.

## 5 CONCLUSION

The overall purpose of this research was to determine the relationship between perceived social support and self‐esteem in burn patients in Sina Hospital of Tabriz. The results of this study showed that high social support increases self‐esteem. Therefore, greater patient support increases self‐esteem and greater adaptation of patients to existing conditions and improves return to life in rehabilitative stages. Accordingly, considering the importance of social support and its generalizability, we suggest that factors affecting social support in burn patients be strengthened.

### 5.1 Clinical implications

Based on the findings of this study, nurses should try to strengthen adaptation and improve the self‐esteem of these patients by establishing appropriate relationships with clients with burn injuries.

## CONFLICTS OF INTEREST

There are no conflicts of interest.

## ETHICAL APPROVAL

This study was approved by the Ethics Committee of IR.IAU.URMIA.REC.1397.20. Informed consent was obtained from participants. The participants were informed about the purpose of the study was voluntary participation, anonymity, confidentiality of information, and the right to withdraw from the study at the desired time.

## Data Availability

All information in the article can be made available to the audience at the Nursing Open discretion.
